# Cardiovascular Risk Factors Predicting Cardiovascular and Cancer Deaths in a Middle-Aged Population Followed-Up for 61 Years until Extinction

**DOI:** 10.3390/jcdd11080240

**Published:** 2024-08-05

**Authors:** Alessandro Menotti, Paolo Emilio Puddu, Paolo Piras

**Affiliations:** 1 Association for Cardiac Research, 00182 Rome, Italy; amenotti2@gmail.com; 2 EA 4650, Signalisation, Électrophysiologie et Imagerie des Lésions D’ischémie Reperfusion Myocardique, Normandie Université, UNICAEN, 14000 Caen, France; 3 Department of Structural Engineering, Sapienza University of Rome, 00185 Rome, Italy; paolo.piras@uniroma3.it

**Keywords:** cancer deaths, cardiovascular risk factors, follow-up until extinction of study population, prediction, Cox models, competing risks, Fine–Gray method

## Abstract

Background and Aim. To study the relationships of cardiovascular risk factors with cancer and cardiovascular mortality in a cohort of middle-aged men followed-up for 61 years. Materials and Methods. A rural cohort of 1611 cancer- and cardiovascular disease-free men aged 40–59 years was examined in 1960 within the Italian Section of the Seven Countries Study, and 28 risk factors measured at baseline were used to predict cancer (*n* = 459) and cardiovascular deaths (*n* = 678) that occurred during 61 years of follow-up until the extinction of the cohort with Cox proportional hazard models. Results. A model with 28 risk factors and cancer deaths as the end-point produced eight statistically significant coefficients for age, smoking habits, mother early death, corneal arcus, xanthelasma and diabetes directly related to events, and arm circumference and healthy diet inversely related. In the corresponding models for major cardiovascular diseases and their subgroups, only the coefficients of age and smoking habits were significant among those found for cancer deaths, to which healthy diet can be added if considering coronary heart disease alone. Following a competing risks analysis by the Fine–Gray method, risk factors significantly common to both conditions were only age, smoking, and xanthelasma. Conclusions. A sizeable number of traditional cardiovascular risk factors were not predictors of cancer death in a middle-aged male cohort followed-up until extinction.

## 1. Introduction

Cardio-oncology is a discipline recently started and rapidly evolving, considering that PUBMED already provides several thousand papers on this topic. The discipline is multi-faceted since it includes a number of different approaches starting from a very wide concept, that is the relationships between cardiovascular diseases (CVDs) and cancer since, in most countries, these two conditions cover around two-thirds or more of all-cause mortality. Actually, many of them are narrative papers insisting on its importance, summarizing others’ findings, and giving suggestions as to how cardio-oncology might be organized from a practical point of view [1,2,3,4,5,6]. Discussed and reported facts frequently deal with largely different areas of research, such as the following: (a) the study of the cardiotoxicity of drugs and, in general, treatments used in cancer therapy [7,8,9,10]; (b) the prediction of cancer in subjects already carrying a major cardiovascular disease (MCVD) [11,12,13,14,15,16]; and (c) the reverse situation where cardiovascular diseases are predicted in subjects with cancer [17,18,19,20,21,22]. Some contributions used existing models created for the prediction of cardiovascular disease to see whether they also predict cancer [23,24,25].

Another area is the search for risk factors and possibly common etiologies in the development of both CVD and overall cancers expressed by personal characteristics, risk factors, and lifestyle behaviors, as well as testing untraditional statistical procedures [26,27,28,29,30,31,32,33,34,35,36,37,38,39,40]. In this area, an analysis variant consists of the creation of a “combined model” that adopts the same set of risk factors and uses as an end-point cancer and CVD events added together [41,42].

The purpose of this article is to contribute to the latter area, that is to analyze, in an epidemiological cohort of middle-aged men, a long list of personal characteristics, frequently considered cardiovascular risk factors, in the attempt to predict in the long-term both overall cancer and MCVD mortality.

## 2. Material and Methods

### 2.1. Population and Measurements

**Figure 1:**
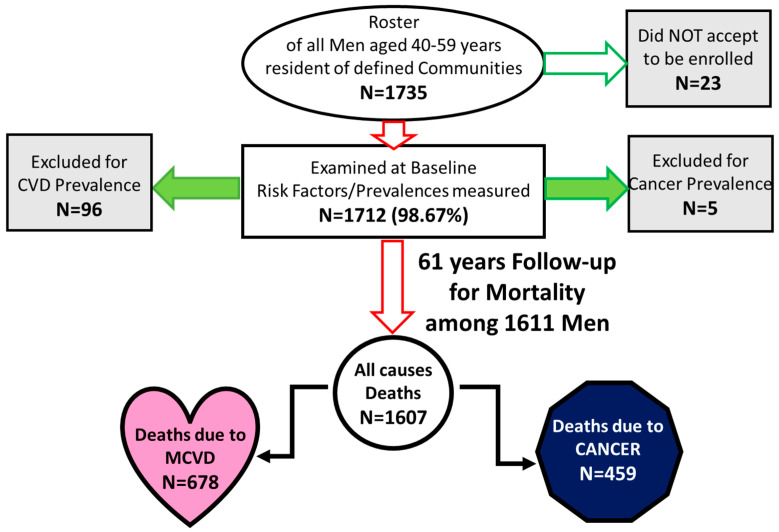
Flow chart of the study.

In 1960, two rural cohorts of middle-aged men (age range 40–59 years) were enrolled within the Italian Section of the Seven Countries Study of Cardiovascular Diseases. A total of 1712 men (Figure 1) were examined (98.67% participation rate), and measurements included demographic, social and behavioral characteristics, cardiovascular risk factors of biophysical, biochemical, and anthropometric nature, clinical diagnosis, and the recording of an electrocardiogram and spirometry testing [43].

Table 1 reports a list of 31 risk factors adopted for this analysis, including units of measurements, notes on procedures, and bibliographic references [44,45,46,47,48,49,50,51].

Follow-up for dates and causes of death was performed for the next 61 years, reaching the practical extinction of the study population. Causes of death were adjudicated by a single reviewer following pre-defined criteria and also exploiting other information from interim examinations, the review of hospital and other clinical records, or interviewing family and hospital doctors and the relatives of the deceased. In fact, we used several information sources since, mainly during the first 3 decades of follow-up, the official causes of death were not very reliable. In the case of multiple causes and uncertainty about the first cause of death, the following rank classification was adopted with violence, cancer, coronary heart disease (CHD), stroke, and other, in that order.

The 8th Revision of the World Health Organization International Classification of Diseases (WHO-ICDs-8) [52] was used for final coding. For cancer deaths, we adopted ICDs-8 codes 140 to 209. For CVD, the problem was more complex since we have repeatedly shown that several characteristics [53] are rather different across different subtypes of CVD and that when pooling them together, the typical characteristics of some of them are diluted or disappear as significant risk factors of the specific form. This fact is surely valid also for the many locations of cancer, but in this analysis, the various locations carried rather small numbers, and therefore their analysis is marginal, keeping all cancer together for the main analysis.

Major cardiovascular diseases mortality were [43,44,45,46,47,48,53,54,55] (A) CHD, including cases with typical syndromes like myocardial infarction, acute ischemic attacks, and sudden coronary death; (B) heart diseases of uncertain etiology (HDUE), including cases with symptomatic heart diseases in the absence of a clear etiology (heart failure, chronic arrhythmia, and blocks), cases classified as hypertensive heart disease (in the absence of evident left ventricle hypertrophy), and cases classified as chronic coronary heart disease in the absence of typical coronary syndromes; (C) cerebrovascular disease, including all kinds of stroke and chronic cerebrovascular conditions (except TIA) (STROKE); and (D) major CVD made by adding the three above conditions (MCVD), thus representing about 96% of all cardiovascular fatal events, then excluding rare diseases or other diseases clearly defined from the etiological point of view.

### 2.2. Statistical Analysis

Mean levels of the selected risk factors were computed for all men and are reported in Table 1, together with units of measurement, notes on methodology and bibliographic references [44,45,46,47,48,49,50,51]. A count was made of cancer deaths by sub-diving them into organ-specific classes limited to groups with at least 10 cases. A similar procedure was run for MCVD events. Cox proportional hazard models were run with 28 risk factors (plus 3 references) as covariates, and having as end-points all cancers, CHD, HDUE, STROKE, and MCVD, separately. Another model, named the combined model, was computed with the same covariates but using the sum of all cancers plus all MCVD as end-points. Similar models were computed with the 5 most common types of cancer as end-points.

ROC (Receiver Operating Characteristic) curves and calibrations were computed for all the above models, including the cross-prediction of cancer using an MCVD model, of MCVD using the cancer model, and, separately, predicting cancer and MCVD using the combined model. The cross-predictions were carried out by keeping the number of events fixed and redistributing them in quintile classes of the predictive-model-estimated probabilities.

A test was made also including, as covariate (yes = 1; 0 = no), the presence of an MCVD as a secondary cause of death (either CHD, STROKE, or HDUE as defined elsewhere [53]) or the presence of cancer as a secondary cause after an MCVD. In each model, prevalent cases with the same diagnosis of that of the end-point were excluded.

A final analysis consisted of running the variant of the Cox model, known as the Fine–Gray method, that allows, through the sub-distribution of risk factors by taking into account the comparison of each pair of them, for the evaluation of the existence of possible competitions. The two conditions challenged in this approach were all cancer deaths and MCVD deaths, while the risk factors were the same as in the Cox models. Two models were produced, i.e., the direct model with MCVD as the principal end-point and cancer as the secondary end-point, and in the inverse model, cancer was the principal end-point and MCVD was the secondary one. Similar methods were used previously, although the primary versus secondary outcomes were different [54,56,57].

## 3. Results

During the 61 years of follow-up among the 1712 men entered in the analysis, 1708 died, 1 was lost to follow-up after 50 years when he was aged 91 years, and 3 were still alive, with their ages ranging from 102 to 106 years. Considering only cancer- and MCVD-free men at entry (n = 1611), during the follow-up, there were 1607 deaths from all causes, 678 deaths due to MCVD, and 459 due to cancer (Figure 1). Principal causes of death classified as cancer and MCVD are reported in Table 2, together with details about the various locations. The first most common location for cancer was stomach, followed by lung, colon–rectum, prostate, and bladder, reflecting a typical situation of the second half of the last century. The first 10 locations (with at least 10 cases) covered 78% of all cases. Among MCVD, CHD was the most common condition, followed by STROKE and HDUE.

The Cox model run for all cancers (Table 3) with 28 risk factors (plus three references) produced eight statistically significant coefficients, i.e., those of age, mother early death, smoking habits, corneal arcus, xanthelasma, and diabetes directly associated with the end-point, while arm circumference and healthy diet were in an inverse fashion.

The model for CHD (Table 4) reflected the usual role of its risk factors with age, systolic blood pressure, and serum cholesterol carrying strong association and significance with events; among the behavioral factors, smoking habits were directly related to cases, while healthy diet and vigorous physical activity were inversely related to events. The model for HDUE (Table 5) was entirely different carrying age, smoking habits, blood pressure, heart rate, and urine protein all directly related to cases, while serum cholesterol, physical activity, and dietary habits had no relationship with events. The model for STROKE (Table 6) included age, smoking habits, blood pressure, and laterality/linearity index (an anthropometric indicator of squatness) directly and strongly related to cases and serum cholesterol moderately so, while vital capacity had an inverse role. Finally, the model of MCVD (Table 7) represented a compromise of the three above models. In fact, age, smoking habits, systolic blood pressure, and laterality/linearity index confirmed their direct role, physical activity also confirmed its inverse role together with vital capacity, while the predictive power of serum cholesterol was diluted slightly, and that of dietary habits was canceled. Another model (not reported in detail) had as an end-point the combined cases of overall cancer and MCVD events (combined model) and provided significant coefficients for 14 out of 28 risk factors.

ROC curves of all the above models were not very good, although four out of six had a significant *p* value, i.e., cancer = 0.545 (*p* = 0.0030); CHD = 0.568 (*p* ≤ 0.001); HDUE = 0.518 (*p* = 0.4342); STROKE = 0.558 (*p* = 0.0039); MCVD = 0.529 (*p* = 0.050); and combined model = 0.509 (*p* = 0.5916).

In a basic Cox model with 28 risk factors and the complete follow-up of 61 years and cancer deaths as the end-point, we forced another variable made by the presence of cancer deaths with a secondary cause of death consisting of any MCVD (59 cases) or the presence of MCVD with cancer as a secondary cause (2 cases). The coefficient was small and not significant. The baseline age was similar and not significantly different versus other cancer patients, while age at death was higher for those with both diseases (77.8 year) than for the others (72.4 years).

Cox models with 28 risk factors were produced also for the five most common cancer locations, with findings summarized here, listing the significant risk factors (full models not reported in details): (i) stomach: age and xanthelasma (both direct association); (ii) lung: age, smoking habits, xanthelasma, baldness (direct associations), and vital capacity (inverse association); (iii) colon–rectum: age, mother early death, body mass index (direct associations), and arm circumference (inverse association); (iv) prostate: age, body mass index, systolic blood pressure, and forced expiratory volume (all direct associations); (v) bladder: none. Some marginally reasonable findings came only from the case of lung cancer and perhaps colon–rectum and prostate, while from the others, any interpretation is hampered by the small numbers involved.

**Figure 2:**
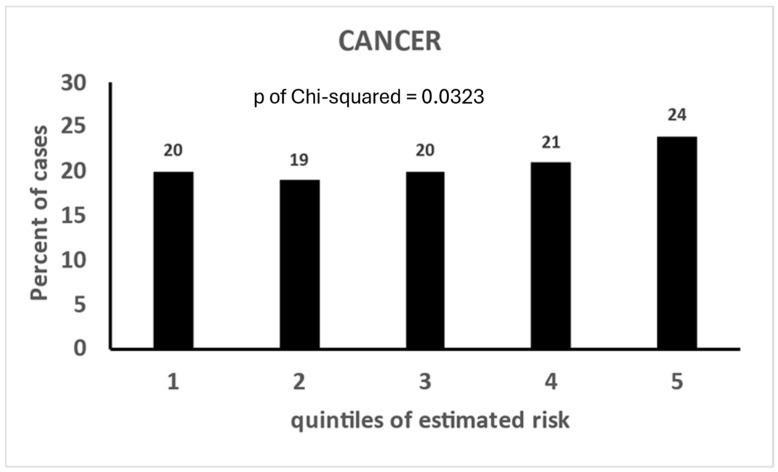
Calibration of Cox model dealing with prediction of all cancers.

**Figure 3:**
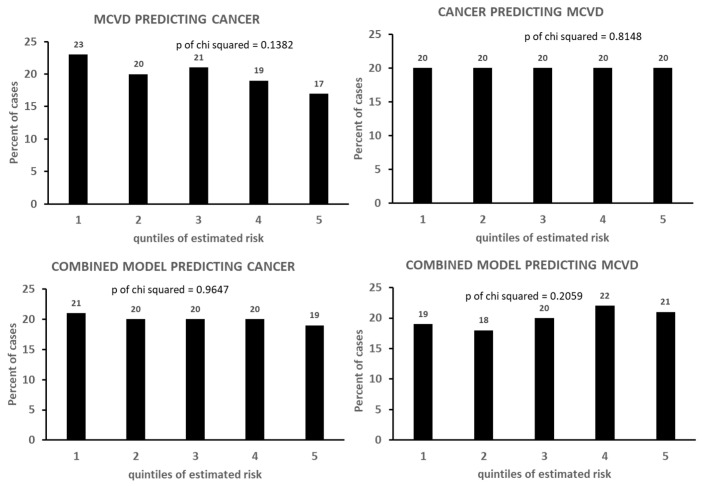
Cross-calibration of cancer and MCVD models and of combined models.

The calibrations of the Cox models dealing only with CVD end-points are given in Table 8, where it appears that only that of CHD is valuable, although those of STROKE and MCVD also provide significant *p* values in the Chi-squared test. The calibrations of the Cox model dealing with cancer or with mixed end-points are given in Figure 2 and Figure 3; only the simple cancer model has a significant *p* value of the Chi-squared test, while all the others are flat or even declining from quintile 1 to quintile 5 (instead of increasing) as for the model of MCVD predicting cancers.

The Fine–Gray models for the evaluation of possible competing risks are reported in Table 9 and Table 10, limiting to a minimum the numerical data in order to facilitate the comparison between the direct and inverse model. In the direct model, where MCVD played the role of the principal end-point, most of the traditional cardiovascular risk factors produced significant coefficients (age, ex-smoker, smokers, laterality/linearity index, systolic blood pressure, serum cholesterol, and xanthelasma with a direct relationship with the end-point, and vigorous physical activity, subscapular skinfold, and vital capacity inversely related to the end-point). Healthy diet was not far from significance since it is related only to CHD, which was not the largest proportion in the pool of MCVD. In the inverse model, where cancers were the principal end-point, risk factors significantly and directly related to the end-point were age, smokers, heart rate, and xanthelasma, while those inversely and significantly related were healthy diet and arm circumference. In summary, risk factors sharing their significance in both end-points were just age, smokers, and xanthelasma.

## 4. Discussion

Among the 28 risk factors and personal characteristics tested in this analysis, only 8 showed a significant relationship with the occurrence of cancer death in a 61-year follow-up. Two of the most typical cardiovascular risk factors, i.e., serum cholesterol and systolic blood pressure, were totally unrelated to these events. On the other hand, two lifestyle behavioral habits usually related to MCVD, i.e., smoking habits and a healthy diet, did so in a clearly opposite and significant way. In particular, the healthy diet hazard ratio (HR) of 0.68 versus the role of the unhealthy diet was associated with an almost one-third difference in cancer mortality risk. Among the other significant risk factors, apart from the expected role of age, corneal arcus and xanthelasma are typical markers of abnormal lipid metabolism and allegedly related to the atherosclerotic process, but they have a very low entry prevalence and are rarely used in similar studies. Mother early death and arm circumference are predictive but somewhat not specific, even for CVD prediction. These two risk factors were always highly significant in models dealing with all-cause mortality and age at death in previous analyses of the same study population [44]. In front of the above findings, those related to CHD, HDUE, and STROKE conditions were expected since they have already been published using somewhat different sets and combinations of risk factors [54]. The major problem was the interpretation of the pooled MCVD since, in that case, a few risk factors were advantaged by the synergistic similar contributions produced in the models by the CVD subtypes, while others were diluted or canceled by the opposite role played in the single subtype models. In particular, the coefficient of serum cholesterol was lower than that of the CHD model because the contribution of HDUE was null from this point of view. Similarly, the protective role of healthy diet seen in the CHD model disappeared because there was no positive contribution from the models of HDUE and STROKE, and, numerically, the cases of CHD became a minority versus the sum of HDUE plus STROKE. As a consequence, a clear comparison of risk factors predicting cancers with those predicting MCVD is distorting the real situation. The best we can say in the comparison of risk factors predicting cancers versus those predicting the pool of MCVD is that only smoking habits play a common direct and significant role. Moreover, if we consider a comparison with the CHD model, healthy dietary habits can be added, as we showed years ago in different types of modeling [45,54,56].

Within the cancer findings, a peculiar direct role is played by corneal arcus and xanthelasma, two indicators of altered lipid metabolism that, conversely, and even more curiously, do not reach statistical significance in any of the CVD models. On the other hand, two of most typical CVD risk factors, i.e., blood pressure and serum cholesterol, do not have significant coefficients in the cancer model.

The performance of the various models was not so good, mainly when cross-calibration was considered. In particular, when an MCVD model tried to predict cancers and a cancer model tried to predict MCVD, the distribution of cases in quintile classes of estimated risk was practically flat (Figure 3). The non-significance of the calibration test and the marginal findings of the ROC curve can partly be explained by the limited power of the significant risk factors and even more by the attrition phenomenon that is heavily influencing the outcome considering the extinction of the study population. In an analysis run on the same material and dealing with the first 15 years of follow-up, in a set of 14 risk factors, largely overlapping with those used here, the only significant ones for cancer deaths were age, smoking habits, and diabetes [55], which correspond in part to those found to be significant in the present analysis. However, when, in the present study, with 61 years of follow-up, competing risks were considered (Table 9 and Table 10), there was only a borderline significance (*p* = 0.07) of diabetes for cancers as primary end-point (inverse model). When the primary end-point was MCVD, diabetes was not significant. To try to explain these results, several aspects should be considered. First, it is possible that either the peculiar combinations of the 28 covariates plus three references selected here or the composition of mortality subsets to form MCVD as an end-point might have contributed. Second, there might be an explanation due to having excluded prevalences (of both MCVD and cancers: Figure 1). Third, after such a long follow-up period, the contribution of diabetes to predict MCVD might have disappeared as those at risk died earlier.

In a previous study assessing competing risks among 10 covariates measured in the same cohorts used here but with 50-year follow-up and with prevalent cases considered [57], diabetes was a significant predictor of CHD deaths when these were joined, by Cox modeling, with all other deaths as a primary end-point. By contrast, when using the Fine–Gray model like we did here, diabetes was never a contributor, apart from the comparisons performed between CHD (primary end-point) and deaths due to violence and peripheral arterial disease (primary end-point) versus CHD deaths [57].

The outcome of the Fine–Gray procedure in the present study for the evaluation of competing risks confirmed, despite some variants, what was shown by the simple Cox models since the only significant risk factors common to both end-points were age, smoking habits, and xanthelasma, which do not correspond to the traditional package of MCVD risk factors and contradicts the claim that cancer and MCVD have the same causality, etiology, determinants, or risk factors, whatever might be the sense or meaning of those possible relationships. The non-significant, marginal coefficient of healthy diet for MCVD in the direct model was due to the abnormal pooling of CHD, HDUE, and STROKE, where that coefficient, usually significant for CHD, was diluted by the presence of the other two subtypes not related to dietary scores in this material.

This analysis suffers from the small size of the study population and the minimal size of the various cancer locations, only partly compensated by the extremely long follow-up of 61 years reaching the extinction of the cohort. Moreover, only men were available in this study. The fact is that, in the middle of the last century, it was estimated that a cohort of women of the same age would have produced a reasonable number of CVD events during the first 10 year only if the size was three-fold the one enrolled among men.

The long list of the tested risk factors includes many of those traditionally employed in CVD epidemiology, although in the past analyses, not necessarily all of them emerged as valuable predictors, or did so only in peculiar risk factor sets or in shorter follow-up periods. We do not claim that our findings should be extrapolated, since several baseline risk factor levels were largely different from the present situation (for example, the high prevalence of smokers). On the other hand, these historic findings might be evaluated from a more general point of view.

The literature somewhat resembling the purpose and structure of our contribution includes some papers where pre-existing models produced from CVD studies were used to predict CVD or cancer events [23,24,25]. The outcome seems valuable, but some uncertainties may arise when a CVD model is used to predict cancer, since it is easy to guess that any model including age and smoking habits might be able to predict cancer events. Instead, one of the quoted papers used polygenic variables that were apparently valuable in predicting both cancer and CVD. Several papers revaluated the role of some anthropometric factors such as body surface, body fat, and waist circumference [29,30,35] that seem to be associated with an excess of both cancers and CVD after having been neglected for decades, except when used as part of metabolic syndrome. However, it is difficult to accept that they play a specific role as risk factors. In an analysis of the NHANES III study, only sedentary and smoking habits were common risk factors for cancer [40], but this is not enough to claim that CVD and cancers have the same determinants or risk factors. On the other hand, the study population was made of CVD patients at risk of possible future cancer. A confirmation of the great role of smoking habits was provided by an analysis dealing with the role of ex-smokers that were prone to developing both conditions [33].

Among isolated risk factors, heart rate [26] and C-reactive protein [37] were found to be directly associated with both CVD and cancer, but the former one is a rather generic risk factor, and the latter is simply an indicator of inflammation that represents a universal physio-pathological mechanism which is common to many types of morbid conditions. On the other hand, clear evidence of the inverse role versus both cancers and some CVD was shown by “healthy diets”, either of Mediterranean or other types [27,28,32], a finding that was shown in our population in 2014 [45] and repeatedly later on when our cohort reached extinction.

Two papers used a combined multivariate model including both cancers and CVD as end-points, and then it was applied separately to the two end-points with good discrimination [40,42]. Unfortunately, these findings cannot compare with a similar approach performed in our analysis because the combined models included a number of clinical details (not available to us and not entirely real risk factors) beyond the common risk factors. Another two papers employed or suggested the use of the competing risk approach to disentangle the real role of the various risk factors between the two end-points [34,41]. One of the two [41] showed that participants of a low risk derived from a combined model had a higher rate of cancer than of CVD, while the opposite happened for participants of a high risk, suggesting that some kind of competition may exist between the two conditions despite the existence of some shared mechanisms in the development of the diseases.

After the consultation of over 2000 papers on the issue, we were unable to find a single contribution similar to ours, where a residential cohort received a number of measurements corresponding to CVD or other types of risk factors, was followed-up long enough to produce a sizeable number of CVD and cancer events, and allowed for the comparison of the predictive role of the same set of CVD (or other type) risk factors on the occurrence of the two diseases. Our study, although limited from several points of view mentioned above, was able to identify smoking habits and partly dietary habits as possible determinants of both cancer and major CVD mortality, while some other factors played different, independent, and generic roles in the two end-points beyond the known, unknown, or unaccounted real causes of the diseases. Claims that CVD risk factor models predict cancer events are probably related to the simple fact that those models include age and smoking habits, whose weight is large enough to play that role. Still, this is not a great discovery and does not justify the surge in an autonomous discipline. This does not exclude that cancer and some CVD may share some common mechanisms in their development, a fact that does not necessarily imply a common causality.

## 5. Conclusions

This analysis suggests that a valuable and typical group of risk factors strongly bound to both cancer and CVD mortality does not exist. What we found is the existence of two behavioral multipotential risk factors, mainly smoking and dietary habits, that may have an additional contribution to the disease’s manifestation beyond the known, unknown, or unaccounted real cause of the disease. The autonomous surge of a discipline, at least in the area whereby risk factors and possibly common etiologies are searched to cooperate in the development of both CVD and overall cancers expressed by personal characteristics, risk factors, and lifestyle behaviors, is not justified.

## Figures and Tables

**Table 1:** Risk factors measured at entry. Definitions, units of measurement, bibliographic, references, and notes.

Risk Factor	Definition or Details	Unit of Measurement	Mean and (SD) or Proportion (%) and (SE)	Bibliographic Reference	Notes
**Age**	Approximated to the nearest birthday	Years	49.1 (5.1)	[44]	
**Father history**	Father dead <65 years from non-infectious nor violent causes	0 = no 1 = yes	21.1 (0.99)%	[44]	From questionnaire
**Mother history**	Mother dead <65 years from non-infectious nor violent causes	0 = no 1 = yes	20.6 (0.98)%	[44]	From questionnaire
**Family history of heart attack**	History of myocardial infarction, or equivalent term in 1st degree siblings	0 = no 1 = yes	37.9 (1.17)%	[44]	From questionnaire
**Marital status**	Presently married (first marriage)	0 = no 1 = yes	90.5 (0.71)%	[44]	From questionnaire
**High socio-economic status** **HSES**	Professional, business, public administrators, foreman, and high-rank clerks	0 = no 1 = yes	11.0 (0.76)%	[44]	
**Sedentary physical activity**	Job-related derived from questions matched with reported occupation	0 = no 1 = yes	9.7 (0.7)%	[45,46]	Dummy reference for physical activity
**Moderate physical activity**	Job-related derived from questions matched with reported occupation	0 = no 1 = yes	22.1 (1.0)%	[45,46]	Classes of physical activity validated by ergonometric procedure and energy intake
**Vigorous physical activity**	Job-related derived from questions matched with reported occupation	0 = no 1 = yes	68.2 (1.1)%	[45,46]	
**Cigarette smoking**	Smokers	0 = no 1 = yes	61.1 (1.2)%	[45]	Dummy reference for smoking habits
**Cigarette smoking**	Ex-smokers	0 = no 1 = yes	13.6 (0.8)%	[45]	
**Cigarette smoking**	Never smokers	0 = no 1 = yes	25.4 (1.1)%	[45]	
**Non-healthy diet**	Dietary history	0 = no 1 = yes	33.4 (1.1)%	[47,48]	Dummy reference for dietary habits
**Intermediate** **Diet**	Dietary history	0 = no 1 = yes	33.3 (1.1)%	[47,48]	Classes of diet derived from factor score of principal component analysis on 18 food groups
**Healthy diet**	Dietary history	0 = no 1 = yes	33.4 (1.1)%	[47,48]	
**Body mass index**	Weight/height squared	kg/m^2^	25.2 (3.7)	[49]	
**Trunk/height ratio**	(sitting height/height) × 100	Ratio	53.3 (1.5)	[49]	
**Shoulder/pelvis shape (ratio)**	Biacromial diameter/bicristal diameter	Ratio	1.36 (0.1)	[49]	
**Laterality/** **linearity index**	(Sum of 2 diameters/height) × 100		40.9 (1.8)	[49]	
**Subscapular skinfold**	Harpenden caliper. Below tip of right scapula	mm	11.8 (5.8)	[49]	
**Midarm circumference**	Right arm. Mathematically cleaned from skin and subcutaneous tissue using the value of tricipital skinfold thickness	mm	268.6 (23.6)	[49,50]	
**Systolic blood pressure**	Supine Average of 2 measurements	mmHg	143.6 (21.0)	[49]	
**Heart rate**	From ECG, average rate in lead I and V_6_	beats/minute	71.3 (12.9)		
**Vital capacity**	Best of 2 tests Adjusted (divided) for height^2^	L/m^2^	1.65 (0.24)	[49]	
**Forced expiratory volume**	Best of 2 tests Adjusted (divided) for height^2^	L/m^2^	1.08 (0.24)	[49]	
**Serum cholesterol**	Method of Abel–Kendall modified by Anderson and Keys. Casual blood sample	mg/dL	201.6 (40.8)	[51]	
**Urine protein**	Spot urines. Semiquantitative method by stix Definite present	0 = absent 1 = present	7.8 (0.6)%		
**Diabetes**	Clinical diagnosis plus spot urine glucose	0 = no 1 = yes	4.7 (0.5)%		
**Baldness**	Partial evident or total	0 = no 1 = yes	29.0 (1.1)%		
**Corneal arcus**	Clinical judgment	0 = no 1 = yes	13.9 (0.8)%		
**Xanthelasma**	Clinical judgment	0 = no 1 = yes	1.5 (0.3)%		

**Table 2:** Cancer deaths in 61 years of follow-up. Details only for locations with at least 10 cases covering 78.2% of all cases.

Cancer Groups	*n* Cases	Proportion % Over All	Notes	*n* Cases after Exclusion of Prevalence
Stomach	81	17.5		81
Lung	78	16.8		77
Colon, rectum	57	12.3	Arbitrarily combined	56
Prostate	50	10.8		49
Bladder	26	5.6		26
Unidentified	20	4.3		19
Pancreas	15	3.2		15
Larynx	14	3.0		14
Brain	11	2.4		11
Liver	11	2.4		11
Others	100	21.6	Covering 31 other locations	100
Total cancer deaths	463	100.0		459
Cardiovascular disease groups	*n* cases	Proportion % over all		
CHD	281	38.7		270
HDUE	216	29.7		206
STROKE	230	31.6		226
Total MCVD deaths	727	100.0		678

See text for acronyms’ explanations.

**Table 3:** Cox proportional hazard model with all cancer deaths as end-point and 28 risk factors (plus 3 references) as covariates. Significance in bold.

Risk Factor	Coefficient	*p* Value	Delta	HR	95% CLs
**Age**	**0.0789**	**<0.0001**	**5**	**1.48**	**1.33 1.65**
High socio-economic status	−0.2167	0.2404	1	0.81	0.56 1.16
Father early death	0.1298	0.2600	1	1.14	0.91 1.43
**Mother early death**	**0.2595**	**0.0239**	**1**	**1.30**	**1.03 1.62**
Familiarity heart attack	−0.0056	0.9547	1	0.99	0.82 1.21
Marriage	0.3029	0.1079	1	1.35	0.94 1.96
Sedentary physical activity	Reference	----	----	----	----
Moderate physical activity	−0.1160	0.5559	1	0.89	0.61 1.31
Vigorous physical activity	−0.0347	0.8534	1	0.97	0.67 1.40
Unhealthy diet	Reference	----	----	----	----
Intermediate diet	−0.2027	0.0937	1	0.82	0.64 1.03
**Healthy diet**	**−0.3790**	**0.0084**	**1**	**0.68**	**0.52 0.91**
Never smoker	Reference	----	----	----	----
Ex-smoker	−0.1307	0.4652	1	0.88	0.62 1.25
**Smoker**	**0.3651**	**0.0017**	**1**	**1.44**	**0.15 1.81**
Body mass index	0.0276	0.3246	3.5	1.10	0.91 1.33
Trunk/height ratio	0.0025	0.9409	1.5	1.00	0.91 1.11
Shoulder pelvis shape	0.9589	0.1413	0.1	1.10	0.97 0.15
Laterality/linearity index	−0.0053	0.8575	1.08	0.99	0.89 1.10
Subscapular skinfold	−0.0222	0.1235	6	0.88	0.74 1.04
**Arm circumference**	**−0.0070**	**0.0150**	**25**	**0.84**	**0.73 0.97**
Systolic blood pressure	0.0010	0.7333	20	1.02	0.91 1.14
Heart rate	0.0069	0.1055	13	1.09	0.98 1.22
Vital capacity	0.3685	0.1673	0.25	1.10	0.96 1.25
Forced expiratory volume	−0.3327	0.1766	0.25	0.92	0.82 1.04
Serum cholesterol	0.0019	0.1133	40	1.08	0.98 1.19
Urine protein	−0.2678	0.2189	1	0.77	0.50 1.17
Baldness	−0.0608	0.5724	1	0.94	0.76 1.16
**Corneal arcus**	**0.3707**	**0.0060**	**1**	**1.45**	**1.11 1.89**
**Xanthelasma**	**1.0177**	**0.0012**	**1**	**2.77**	**1.49 5.12**
**Diabetes**	**0.4358**	**0.0448**	**1**	**1.55**	**1.01 2.37**

HR = hazard ratio; 95% CLs = confidence limits. Delta for computation of HR roughly equal to the standard deviation for continuous variables. For units of measurement see Table 1.

**Table 4:** Cox proportional hazard model with CHD deaths as end-point and 28 risk factors (plus 3 references) as covariates. Significance in bold.

Risk Factor	Coefficient	*p* Value	Delta	HR	95% CLs
**Age**	**0.0573**	**<0.0001**	**5**	**1.33**	**1.16 1.53**
High socio-economic status	−0.2655	0.2050	1	0.77	0.51 1.16
Father early death	−0.1229	0.4316	1	0.88	0.65 1.20
Mother early death	0.1745	0.2440	1	1.19	0.89 1.60
Familiarity heart attack	0.1503	0.2319	1	1.16	0.91 1.49
Marriage	−0.2924	0.1340	1	0.75	0.51 1.09
Sedentary physical activity	Reference	----	----	----	----
**Moderate physical activity**	**−0.4563**	**0.0275**	**1**	**0.63**	**0.42 0.95**
**Vigorous physical activity**	**−0.6661**	**0.0009**	**1**	**0.51**	**0.35 0.76**
Unhealthy diet	Reference	----	----	----	----
**Intermediate diet**	**−0.3743**	**0.0162**	**1**	**0.69**	**0.51 0.93**
**Healthy diet**	**−0.4787**	**0.0099**	**1**	**0.62**	**0.43 0.89**
Never smoker	Reference	----	----	----	----
Ex-smoker	0.1093	0.5968	1	1.12	0.74 1.67
**Smoker**	**0.2870**	**0.0514**	**1**	**1.33**	**1.00 1.78**
Body mass index	0.0039	0.9102	3.5	1.01	0.80 1.28
Trunk/height ratio	0.0014	0.9737	1.5	1.00	0.88 1.14
Shoulder pelvis shape	−0.2723	0.7438	0.1	0.97	0.83 1.15
Laterality/linearity index	0.0377	0.3394	1.8	1.07	0.93 1.23
Subscapular skinfold	−0.0151	0.3714	6	0.91	0.75 1.11
Arm circumference	−0.0034	0.3493	25	0.92	0.77 1.10
**Systolic blood pressure**	**0.0137**	**<0.0001**	**20**	**1.32**	**1.16 1.49**
Heart rate	−0.0038	0.4714	13	0.95	0.83 1.09
Vital capacity	−0.5223	0.1277	0.25	0.88	0.74 1.04
Forced expiratory volume	−0.4094	0.2069	0.25	0.90	0.77 1.04
**Serum cholesterol**	**0.0062**	**<0.0001**	**40**	**1.28**	**1.14 1.45**
Urine protein	0.0749	0.7487	1	1.08	0.68 1.70
Baldness	0.1835	0.1607	1	1.20	0.93 1.55
Corneal arcus	0.0606	0.7464	1	1.06	0.74 1.53
Xanthelasma	0.6478	0.1630	1	1.91	0.77 4.75
Diabetes	0.1282	0.6628	1	1.14	0.64 2.02

HR = hazard ratio; 95% CLs = confidence limits. Delta for computation of HR roughly equal to the standard deviation for continuous variables. For units of measurement see Table 1.

**Table 5:** Cox proportional hazard model with HDUE deaths as end-point and 28 risk factors (plus 3 references) as covariates. Significance in bold.

Risk Factor	Coefficient	*p* Value	Delta	HR	95% CLs
**Age**	**0.1674**	**<0.0001**	**5**	**2.31**	**1.94 2.74**
High socio-economic status	−0.0849	0.7504	1	0.92	0.54 1.55
Father early death	0.2831	0.0895	1	1.33	0.96 1.84
Mother early death	0.2658	0.1179	1	1.30	0.93 1.82
Familiarity heart attack	0.0839	0.5636	1	1.09	0.82 1.45
Marriage	0.0386	0.8803	1	1.04	0.63 1.72
Sedentary physical activity	Reference	----	----	----	----
Moderate physical activity	−0.2207	0.4299	1	0.80	0.46 1.39
Vigorous physical activity	−0.3741	0.1596	1	0.69	0.41 1.16
Unhealthy diet	Reference	----	----	----	----
Intermediate diet	0.1184	0.5443	1	0.89	0.61 1.30
Healthy diet	0.0309	0.8857	1	1.03	0.68 1.57
Never smoker	Reference	----	----	----	----
Ex-smoker	0.3568	0.1205	1	1.43	0.91 2.24
**Smoker**	**0.5073**	**0.0034**	**1**	**1.66**	**1.18 2.33**
Body mass index	−0.0247	0.5656	3.5	0.92	0.68 1.23
Trunk/height ratio	0.0866	0.0894	1.5	1.14	0.98 1.32
Shoulder pelvis shape	0.2434	0.8025	0.1	1.02	0.85 1.24
Laterality/linearity index	0.0624	0.1565	1.8	1.12	0.96 1.31
Subscapular skinfold	−0.0096	0.6421	6	0.94	0.74 1.20
Arm circumference	−0.0055	0.2255	25	0.87	0.70 1.09
**Systolic blood pressure**	**0.0145**	**0.0003**	**20**	**1.34**	**1.14 1.56**
**Heart rate**	**−0.0150**	**0.0257**	**13**	**0.82**	**0.39 0.98**
Vital capacity	−0.0992	0.8060	0.25	0.98	0.80 1.19
Forced expiratory volume	−0.4286	0.2439	0.25	0.90	0.75 1.08
Serum cholesterol	0.0014	0.4680	40	1.06	0.91 1.22
**Urine protein**	**0.5421**	**0.0311**	**1**	**1.72**	**1.05 2.81**
Baldness	0.1374	0.3721	1	1.15	0.85 1.55
Corneal arcus	0.2371	0.2479	1	1.32	0.85 1.89
Xanthelasma	0.6092	0.4005	1	1.81	0.44 7.61
Diabetes	0.3401	0.3362	1	1.41	0.70 2.81

HR = hazard ratio; 95% CLs = confidence limits. Delta for computation of HR roughly equal to the standard deviation for continuous variables. For units of measurement see Table 1.

**Table 6:** Cox proportional hazard model with STROKE deaths as end-point and 28 risk factors (plus 3 references) as covariates. Significance in bold.

Risk Factor	Coefficient	*p* Value	Delta	HR	95% CLs
**Age**	**0.1110**	**<0.0001**	**5**	**1.74**	**1.49 2.04**
High socio-economic status	0.1765	0.4284	1	1.19	0.77 1.85
Father early death	0.1379	0.3992	1	1.15	0.83 1.58
Mother early death	0.0904	0.5895	1	1.09	0.79 1.52
Familiarity heart attack	0.1033	0.4546	1	1.11	0.85 1.45
Marriage	−0.1881	0.4075	1	0.83	0.53 1.29
Sedentary physical activity	Reference	----	----	----	----
Moderate physical activity	−0.1058	0.6736	1	0.90	0.55 1.47
Vigorous physical activity	−0.3466	0.1698	1	0.71	0.43 1.16
Unhealthy diet	Reference	----	----	----	----
Intermediate diet	0.0210	0.9074	1	1.02	0.72 1.46
Healthy diet	−0.0344	0.8686	1	0.97	0.64 1.45
Never smoker	Reference	----	----	----	----
**Ex-smoker**	**0.5446**	**0.0099**	**1**	**1.72**	**1.14 2.61**
**Smoker**	**0.3625**	**0.0317**	**1**	**1.44**	**1.03 2.00**
Body mass index	−0.0484	0.2267	3.5	0.84	0.64 1.11
Trunk/height ratio	0.0711	0.1455	1.5	1.11	0.96 1.28
Shoulder pelvis shape	0.1870	0.8453	0.1	1.02	0.84 1.23
**Laterality/linearity index**	**0.1157**	**0.0074**	**1.8**	**1.23**	**1.06 1.43**
Subscapular skinfold	−0.0121	0.5247	6	0.93	0.74 1.16
Arm circumference	−0.0007	0.8709	25	0.98	0.80 1.21
**Systolic blood pressure**	**0.0126**	**0.0007**	**20**	**1.29**	**1.11 1.49**
Heart rate	0.0025	0.6717	13	1.03	0.89 1.20
**Vital capacity**	**−0.0136**	**0.0106**	**0.25**	**0.78**	**0.64 0.94**
Forced expiratory volume	0.3464	0.3557	0.25	1.09	0.91 1.31
**Serum cholesterol**	**0.0034**	**0.0506**	**40**	**1.15**	**1.00 1.31**
Urine protein	0.2200	0.3901	1	1.25	0.75 2.06
Baldness	0.1434	0.3235	1	1.15	0.87 1.53
Corneal arcus	0.2574	0.1882	1	1.29	0.88 1.90
Xanthelasma	0.4620	0.4380	1	1.59	0.49 5.10
Diabetes	0.4972	0.0966	1	1.64	0.91 2.96

HR = hazard ratio; 95% CLs = confidence limits. Delta for computation of HR roughly equal to the standard deviation for continuous variables. For units of measurement see Table 1.

**Table 7:** Cox proportional hazard model with MCVD deaths as end-point and 28 risk factors (plus 3 references) as covariates. Significance in bold.

Risk Factor	Coefficient	*p* Value	Delta	HR	95% CLs
**Age**	**0.1047**	**<0.0001**	**5**	**1.69**	**1.55 1.84**
High socio-economic status	−0.0632	0.6315	1	0.94	0.73 1.22
Father early death	0.0760	0.4145	1	1.08	0.90 1.30
Mother early death	0.1745	0.0608	1	1.19	0.99 1.43
Familiarity heart attack	0.1134	0.1470	1	1.12	0.96 1.31
Marriage	−0.1685	0.1877	1	0.84	0.66 1.09
Sedentary physical activity	reference	----	----	----	----
**Moderate physical activity**	**−0.2919**	**0.0342**	**1**	**0.75**	**0.57 0.98**
**Vigorous physical activity**	**−0.5052**	**0.0002**	**1**	**0.60**	**0.46 0.78**
Unhealthy diet	Reference	----	----	----	----
Intermediate diet	−0.1945	0.0529	1	0.82	0.68 1.00
Healthy diet	−0.2000	0.0829	1	0.82	0.65 1.03
Never smoker	Reference	----	----	----	----
**Ex-smoker**	**0.3261**	**0.0083**	**1**	**1.39**	**1.09 1.77**
**Smoker**	**0.3758**	**0.0001**	**1**	**1.46**	**1.21 1.75**
Body mass index	−0.0205	0.3562	3.5	0.93	0.80 1.08
Trunk/height ratio	0.0466	0.0897	1.5	1.07	0.99 1.16
Shoulder pelvis shape	0.0604	0.9089	0.1	1.01	0.91 1.12
**Laterality linearity index**	**0.0726**	**0.0028**	**1.8**	**1.14**	**1.05 1.24**
Subscapular skinfold	−0.0135	0.2109	6	0.92	0.81 1.05
Arm circumference	−0.0031	0.1833	25	0.93	0.82 1.04
**Systolic blood pressure**	**0.0137**	**<0.0001**	**20**	**1.32**	**1.21 1.43**
Heart rate	−0.0048	0.1523	13	0.94	0.86 1.02
**Vital capacity**	**−0.5499**	**0.0117**	**0.25**	**0.87**	**0.78 0.97**
Forced expiratory volume	−0.1667	0.3856	0.25	0.96	0.87 1.06
**Serum cholesterol**	**0.0040**	**<0.0001**	**40**	**1.17**	**1.09 1.27**
Urine protein	0.2571	0.0716	1	1.29	0.98 1.71
Baldness	0.1551	0.0587	1	1.17	0.99 1.37
Corneal arcus	0.1876	0.0963	1	1.21	0.97 1.50
Xanthelasma	0.5740	0.0784	1	1.78	0.94 3.36
Diabetes	0.3028	0.0926	1	1.35	0.95 1.93

HR = hazard ratio; 95% CLs = confidence limits. Delta for computation of HR roughly equal to the standard deviation for continuous variables. For units of measurement see Table 1.

**Table 8:** Calibration of Cox models for CVD end-points with cases (expressed as percent of all cases) distributed in quintile classes of estimated risk.

End-Points of Models	Quintiles of Estimated Risk					*p* of Chi-Squared
	1	2	3	4	5	
**CHD**	15	16	20	23	26	0.0099
**HDUE**	19	18	19	21	23	0.7370
**STROKE**	17	14	20	25	24	0.0428
**MCVD**	18	18	20	22	22	0.0407

See text for acronyms’ explanations.

**Table 9:** Competing risks analysis following the Fine–Gray method: coefficients and *p* values. Significance in bold.

Risk Factor	Direct Model		Inverse Model	
	Coefficient	*p* Value	Coefficient	*p* Value
**Age**	**0.1026**	**<0.0001**	**0.0804**	**<0.0001**
High socio-economic status	−0.2382	0.0550	**−0.3672**	**0.0440**
Father early death	0.0442	0.6400	0.0562	0.6400
Mother early death	0.1190	0.1900	0.1664	0.1600
Familiarity heart attack	0.0776	0.3100	−0.0732	0.4700
Marriage	−0.1304	0.3400	0.3288	0.0700
Sedentary physical activity	Reference	----	Reference	----
Moderate physical activity	−0.1297	0.3800	0.0729	0.7100
Vigorous physical activity	**−0.3949**	**0.0044**	−0.2067	0.2700
Unhealthy diet	Reference	----	Reference	----
Intermediate diet	−0.1506	0.1200	−0.1925	0.1200
Healthy diet	−0.1898	0.0990	**−0.3592**	**0.0170**
Never smoker	Reference	----	Reference	----
Ex-smoker	**0.4620**	**0.0001**	−0.0072	0.9700
**Smoker**	**0.3930**	**<0.0001**	**0.4074**	**<0.0001**
Body mass index	−0.0126	0.5900	0.0419	0.1700
Trunk/height ratio	0.0319	0.2300	−0.0156	0.6600
Shoulder pelvis shape	0.2554	0.5900	1.0524	0.1300
Laterality linearity index	**0.0665**	**0.0052**	−0.0328	0.3100
Subscapular skinfold	**−0.0221**	**0.0410**	−0.0221	0.1500
Arm circumference	−0.0041	0.0820	**−0.0083**	**0.0055**
Systolic blood pressure	**0.0146**	**<0.0001**	0.0010	0.7500
Heart rate	0.0055	0.1200	**0.0167**	**0.0001**
Vital capacity	**−0.7609**	**0.0008**	0.2246	0.4000
Forced expiratory volume	−0.0914	0.6500	−0.3103	0.2000
Serum cholesterol	**0.0037**	**0.0001**	0.0020	0.1200
Urine protein	0.2002	0.1800	−0.2769	0.2200
Baldness	0.1379	0.0860	−0.1041	0.3600
Corneal arcus	0.0398	0.7400	0.2548	0.0680
**Xanthelasma**	**0.7281**	**0.0200**	**1.1972**	**0.0001**
Diabetes	0.1883	0.4100	0.3409	0.0770

In the direct model, MCVD mortality is the principal end-point, and cancer mortality is the secondary end-point. In the inverse model, cancer mortality is the principal end-point, and MCVD mortality is the secondary end-point.

**Table 10:** Competing risks analysis following the Fine–Gray method: deltas and hazard rates. Significance in bold.

Risk Factor	Delta	Direct Model		Inverse Model	
		HR	95% CLs	HR	95% CLs
**Age**	**1**	**1.67**	**1.52 1.83**	**1.49**	**1.33 1.68**
High socio-economic status	1	0.76	0.58 1.01	**0.69**	**0.48 0.99**
Father early death	1	1.05	0.87 1.26	1.06	0.84 1.33
Mother early death	1	1.13	0.94 1.34	1.18	0.94 1.49
Familiarity heart attack	1	1.08	0.93 1.26	0.93	0.76 1.13
Marriage	1	0.88	0.67 1.15	1.39	0.97 1.98
Sedentary physical activity	Reference	----	----	----	----
Moderate physical activity	1	0.88	0.66 1.17	1.08	0.74 1.57
Vigorous physical activity	1	**0.67**	**0.51 0.88**	1.23	0.85 1.78
Unhealthy diet	Reference	----	----	----	----
Intermediate diet	1	0.86	0.71 1.04	0.82	0.65 1.05
Healthy diet	1	0.83	0.66 1.04	**0.70**	**0.52 0.94**
Never smoker	Reference	----	----	----	----
Ex-smoker	1	**1.59**	**1.26 2.00**	0.99	0.70 1.41
**Smoker**	1	**1.48**	**1.24 1.77**	**1.50**	**1.19 1.89**
Body mass index	3.5	0.96	0.81 1.12	1.16	0.94 1.43
Trunk/height ratio	1.5	1.05	0.97 1.14	0.98	0.88 1.08
Shoulder pelvis shape	0.1	1.03	0.94 1.12	1.11	0.97 1.27
Laterality linearity index	1.8	**1.13**	**1.04 1.23**	0.94	0.84 1.06
Subscapular skinfold	6	0.88	0.77 0.99	0.88	0.73 1.05
Arm circumference	25	0.90	0.80 1.01	**0.81**	**0.70 0.94**
Systolic blood pressure	20	**1.34**	**1.22 1.47**	1.02	0.91 1.15
Heart rate	13	1.07	0.98 1.18	**1.24**	**1.11 1.39**
Vital capacity	0.25	0.83	0.74 0.92	1.06	0.93 1.20
Forced expiratory volume	0.25	0.98	0.88 1.08	0.93	0.82 1.04
Serum cholesterol	40	**1.16**	**1.08 1.25**	1.08	0.98 1.20
Urine protein	1	1.22	0.91 1.63	0.76	0.49 1.18
Baldness	1	1.15	0.98 1.34	0.90	0.72 1.12
Corneal arcus	1	1.04	0.82 1.32	1.29	0.98 1.70
**Xanthelasma**	1	**2.07**	**1.12 3.82**	**3.31**	**1.81 6.06**
Diabetes	1	1.21	0.77 1.88	1.14	0.96 20.5

HR = hazard ratio; 95% CLs = confidence limits. Delta for computation of HR roughly equal to the standard deviation for continuous variables. For units of measurement see Table 1.

## Data Availability

The data and computing codes are not available for replication because the original data are not publicly available, although the Board of Directors of the Study may evaluate specific requests for dedicated analyses.
